# The Intermediary Metabolism of 3: 4-Benzpyrene: The Biosynthesis and Identification of the X_1_ and X_2_ Metabolites

**DOI:** 10.1038/bjc.1958.73

**Published:** 1958-12

**Authors:** K. H. Harper


### THE INTERMEDIARY METABOLISM OF 3 : 4-BENZPYRENE : THE BIOSYNTHESIS AND IDENTIFICATION OF THE $X_1$ AND $X_2$ METABOLITES

K. H. HARPER

*From the Department of Cancer Research, Mount Vernon Hospital and the Radium Institute, Northwood, Middlesex*

Received for publication October 24, 1958

A PREVIOUS study of the intermediary metabolism of 3 : 4-benzpyrene has been reported (Harper, 1958*b*). The $X_1$ metabolite ($BPX_1$) was considered to be the glucuronide conjugate of $F_1$, to which a free phenolic nature was assigned, and evidence was obtained that 8-benzpyrenol is also eliminated in conjugated form in the bile. The $X_2$ metabolite ($BPX_2$) was not identified but its absorption spectrum was considered to be more in keeping with a fully aromatic benzpyrenoid configuration rather than the dihydroxy-dihydro structure proposed by Weigert and Mottram (1946).

Conjugation with glucuronic and sulphuric acids is known to be a general process for the detoxification of phenols. It seemed hopeful therefore that reintroduction of 8-benzpyrenol and the $F_1$ phenol into the animal body would lead to their elimination in the bile or urine in conjugation with these two acids. These conjugates could then be used as reference compounds for the fractionation and subsequent identification of the intermediate metabolites of 3 : 4-benzpyrene.

### MATERIALS AND METHODS

8-Benzpyrenol was synthesised by the method of Cook, Ludwiczak and Schoental (1950). Final purification was effected by chromatography on alumina from benzene followed by crystallisation from benzene-light petroleum. The resulting yellow needles melted at 225–228° C. (decomp.) and spectroscopically appeared almost identical to 8-benzpyrenol of metabolic origin (Berenblum, Crowfoot, Holiday and Schoental, 1943; Weigert and Mottram, 1946).

The absorption maxima (in ethanol) were at 228, 259, 268, 293–294, 308, 346, 362, 380·5, 402 and 426 m$\mu$. There is a slight displacement of these figures from those reported by Conney, Miller and Miller (1957) for synthetic 8-benzpyrenol in ethanol.

The phenol was prepared for injection as a fine colloidal dispersion containing 0·5 mg. per c.c. by dissolving it in a small volume of acetone and injecting the acetone solution beneath the surface of glass double-distilled water. The acetone was then pumped off *in vacuo*. The mice (Strong A strain) in batches of 10–15 received an intravenous injection of 0·5 c.c. of this colloid and were then killed at intervals of 1–4 hours.

The $F_1$ phenol was obtained by acid hydrolysis of pooled $X_1$ metabolite isolated from the small intestines of mice which had been injected with 3 : 4-benzpyrene. It was prepared as a colloidal dispersion as described for 8-benzpyrenol and this was used for the injection of four mice.

After the desired time interval the particular organ to be examined was quickly dissected out and subjected to an extraction procedure similar to that described for the intermediate metabolites of 3 : 4-benzpyrene. This involved initial extraction with 70 per cent acetone, removal of the acetone *in vacuo* and saturation of the aqueous phase with ammonium sulphate. After repeated extraction with xylene the aqueous phase was then acidified with hydrochloric acid and re-extracted with xylene. This resulted in the transference of all blue fluorescent material into the xylene phases. These were then combined and chromatographed on columns of silica gel for the removal of possible X-type derivatives. Free phenol and quinone in the filtrate from this adsorbent were detected by chromatography on alumina.

All descriptions of fluorescence refer to the appearance under ultra-violet light.

$\beta$-Glucuronidase (bacterial) and *p*-nitrophenylsulphate were obtained from the Sigma Chemical Company, Missouri, potassium hydrogen saccharate from George T. Gurr Ltd., silica gel (100/200 mesh), adenylic acid, $\alpha$-ketoglutaric acid and adenosinetriphosphate (ATP) from L. Light and Co. Ltd. and alumina from B.D.H. Ltd. Takadiastase was a gift from Parke Davis, Co. Ltd.

All organic reagents were obtained free from fluorescence by fractional distillation.

### RESULTS

### *Metabolism of* 8-*benzpyrenol*

The appearance of the opened abdominal cavity under ultra-violet light was very similar to that arising from the intravenous injection of the parent hydrocarbon, 3 : 4-benzpyrene. A strong blue fluorescence was associated with the gall bladder, gut and kidney but the weak fluorescence of the bladder suggested that little excretion of fluorescent metabolities was taking place in the urine.

(a) *Duodenum and small intestine.*—Chromatography of the xylene extract on silica gel yielded a green coloured, strong blue/white fluorescent zone at the surface. On development with amyl alcohol a strong blue fluorescent fraction passed down the column and was collected in the filtrate, the initial yellow non-fluorescent fraction being discarded.

The amyl alcohol filtrate was diluted with 10 times its own volume of petroleum ether (b.p. 40–60° C.) and rechromatographed on silica gel. The bright blue fluorescent metabolite was held as an almost colourless zone at the surface from which it was readily eluted with ethanol. The absorption spectrum of the eluate was similar to, but not identical with, the $X_1$ metabolite derived from 3 : 4-benzpyrene (Fig. 1, Table I).

After development with amyl alcohol the whole of the original silica gel column possessed a blue/white fluorescence, this being most intense in the green coloured surface zone. On elution with ethanol this surface zone yielded a pale green solution with an intense blue fluorescence. Its absorption spectrum was similar to that of $X_2$ derived from 3 : 4-benzpyrene but it obviously contained strongly absorbing background material. Partial purification was effected by transfer to water, acidification with hydrochloric acid and extraction with ether. The metabolite passed out in the ether phase and possessed the spectrum shown (Fig. 2, Table I).

FIG. 1.—Absorption spectra in ethyl alcohol. 1. $X_1$ metabolite of 3 : 4-benzpyrene (upper scale). 2. $X_1$ metabolite of 8-benzpyrenol (lower scale).

A trace amount only of free 8-benzpyrenol was detected on the alumina chromatogram.

TABLE I

| | Absorption maxima in ethanol—mμ | | | | | | | | | |
|---|---|---|---|---|---|---|---|---|---|---|
| $BPX_1$ . . | 256 | 266 | — | 286 | 298 | 363 | 369 | 379 | 388 | 408–409 |
| 8-OH.$X_1$ . | 256–257 | 268 | 279 | 289 | 302 | 362 | 369 | 381 | 388 | 409 |
| $F_1X_1$ . . | — | — | — | — | 298 | 362 inf. | 369 inf. | 378–380 | — | 406–410 inf. |
| $BPX_2$ . . | 256–260 | 268 | — | 287 | 298 | 362–364 | — | 380 | 392 | 414 |
| 8-OH.$X_2$ . | 256 | 268 | — | 290 | 303 | 362 | — | 381 | 392 | 416–417 |

$BPX_1$ and $BPX_2$ = $X_1$ and $X_2$ metabolites of 3 : 4-benzpyrene.
8-OH.$X_1$ and 8-OH.$X_2$ = $X_1$ and $X_2$ metabolites of 8-benzpyrenol.
$F_1X_1$ = $X_1$ Metabolite of the $F_1$-phenol.

(b) *Caecum and large intestine.*—Extracts from animals killed 1 hour after injection yielded only small amounts of the X-type derivatives and free 8-benzpyrenol. The amount of free phenol increased progressively with time and a considerable quantity was present 4 hours after injection.

(c) *Bile.*—Extraction of the bright blue fluorescent bile with ether removed little fluorescent material but after acidification an ether extract possessed a bright blue fluorescence. After transfer to xylene and chromatography on silica gel followed by alumina $X_1$- and $X_2$-type derivatives were identified on the silica gel and free 8-benzpyrenol on the alumina. The extraction of free phenol only after acidification suggested its formation from an acid-labile precursor. This factor will be referred to later.

(d) *Kidney.*—Appreciable amounts of both $X_1$- and $X_2$-type derivatives were obtained from this tissue.

(e) *Bladder.*—A small amount of 8-benzpyrenol was extractable directly from the urine by ether. Only small amounts of the X-type derivatives and free phenol were extracted after acidification.

(f) *Liver.*—Little blue fluorescent material was extracted with 70 per cent acetone and the $X_1$-type derivative only was identified with any certainty.

FIG. 2.—Absorption spectra in ethyl alcohol. 1. $X_2$ metabolite of 3 : 4-benzpyrene (upper scale). 2. $X_2$ metabolite of 8-benzpyrenol (lower scale).

*Properties and identification of the metabolites*

The physical and chemical properties of the two derivatives closely paralleled those previously reported for the X-metabolites of 3 : 4-benzpyrene (Weigert and Mottram, 1946; Harper, 1958*b*).

Both were adsorbed on silica gel from xylene and the $X_1$-type only was eluted with amyl alcohol. The $X_2$-type was associated with the coloured surface zone of the chromatogram and was only partially eluted with ethanol. Better elution was effected with either 1 : 1 ethanol-pH 6 phosphate buffer or ethanol containing a few drops of hydrochloric acid.

Both were strongly adsorbed on alumina and the $X_1$-type only was partially removed by ethanol.

Both were water soluble although a certain amount of insoluble matter remained on evaporating the ethanolic eluates to dryness.

On gently warming in 0·1 N-HCl under nitrogen the $X_1$-type was rapidly converted to 8-benzpyrenol whereas the $X_2$-type remained unaffected. With stronger acid (10 N-HCl) and heating the solution at 100° C. for 2 hours under nitrogen the $X_2$-type was also converted to 8-benzpyrenol.

Further investigation of the hydrolytic conditions revealed that, after the addition of acid to an aqueous solution of the $X_1$-type metabolite, although there was no apparent change in its absorption spectrum, a considerable amount of free 8-benzpyrenol was immediately extractable with ether. It appeared therefore that the metabolite was instantly decomposed by the combined effect of acid and ether.

The most likely explanation for this behaviour was that the metabolites were sulphate and glucuronide conjugates of 8-benzpyrenol for, in an analogous study of the metabolism of 1-naphthol (Boyland and Wiltshire, 1953), the phenol was excreted either as free naphthol, naphthylsulphuric acid or naphthylglucuronide and only a relatively small amount was degraded by metabolic processes in the animal body. The evidence thus far pointed towards the $X_1$-type derivative being the sulphate and the $X_2$-type the glucuronide for phenylglucuronides are, in general, more resistant to acid hydrolysis than are the sulphates (Bray and Thorpe, 1954) and, in the case of steroid conjugates, the glucuronides are more strongly adsorbed on alumina than are the sulphates (Barlow, 1957).

Experiments were therefore carried out to test this hypothesis.

*Test for the glucuronide moiety*

Solutions of the metabolites in 2 c.c. water (estimated as containing as much as 25 $\gamma$) were heated with 2 c.c. 0·375 per cent naphthoresorcinol reagent and 4 c.c. technical hydrochloric acid at 100° C. for 2 hours. The mixtures were then cooled and extracted with 10 c.c. ether. The ether extract of the $X_1$-type was pale yellow in colour although a reddish tinge was given by impure samples of the metabolite contaminated by yellow colouring matter. The $X_2$-type invariably yielded a reddish/blue colour.

Although not conclusive this suggested a glucuronide moiety in the $X_2$-type derivative.

*Action of β-glucuronidase*

Detailed studies of the enzyme β-glucuronidase and of its inhibition by saccharo-1 : 4-lactone have been made by Levvy and his co-workers (Levvy, 1948, 1952; Karunairatnam and Levvy, 1949) and the statement is made (Conchie and Levvy, 1957) that saccharo-1 : 4-lactone as such, or in the form of a saccharate solution, has been shown to inhibit β-glucuronidase from every source that has been studied.

Incubations of the metabolites with β-glucuronidase were carried out as described in the Sigma Bulletin No. 105 using a 1 per cent solution of the enzyme. In control experiments boiled 1 per cent solutions were used. The incubates were then cooled and extracted with ether.

The extracts from the control and $X_1$-type incubates were only weakly fluorescent whereas that from the $X_2$-type was bright blue and exhibited the characteristic spectrum of 8-benzpyrenol.

In the presence of $10^{-2}$ M boiled saccharate solution the hydrolytic effect of the enzyme on the $X_2$-type metabolite was almost completely abolished.

This result indicated a β-glucuronosidic linkage in the $X_2$-type derivative only.

*Action of takadiastase*

A detailed investigation of the arylsulphatase activity of takadiastase was made by Robinson, Smith, Spencer and Williams (1952).

The optimum pH for arylsulphatase activity appears to be about 6 but in the present experiments acetate buffer pH 6·8 was used in order to eliminate possible pH effect on the metabolites. Under these conditions the takadiastase still possessed appreciable activity towards *p*-nitrophenylsulphate as substrate but the activity was bolished on boiling the enzyme solution for 15 minutes.

Incubations were carried out as described by Robinson *et al.* using the boiled enzyme as control. The incubates were then cooled and extracted with ether.

The extracts from the control and $X_2$-type incubates were only weakly fluorescent whereas that from the $X_1$-type was bright blue and exhibited the characteristic spectrum of 8-benzpyrenol.

An arylsulphate linkage was thus indicated in the $X_1$-type metabolite only.

*Liver slice experiments*

The conjugation of phenols with sulphuric and glucuronic acids in isolated liver slices has been reported by Storey (1950), De Meio and Tkacz (1952).

Fifteen grammes mouse liver slices were incubated in 50 c.c. Tyrode buffer containing 1 mg. 8-benzpyrenol with oxygenation. The slices soon assumed a bright blue fluorescence and after 2 hours were extracted as described for the *in vivo* tissue.

Appreciable amounts of both $X_1$- and $X_2$-type derivatives and benzpyrene-5 : 8-quinone were identified on the chromatograms in addition to unchanged 8-benzpyrenol.

*Glucuronide synthesis in liver homogenate*

The failure of liver homogenates to synthesise glucuronides was studied by Dutton and Storey (1954). They found that glucuronide synthesis could be restored by the addition of an extract of boiled liver to the homogenate and subsequent work led to the identification of the essential factor as being uridine disphosphate glucuronic acid (UDPGA) (Storey and Dutton, 1955).

An investigation of the metabolism of 8-benzpyrenol in mouse liver homogenate was first made under the conditions described for liver slices. Under these conditions a small amount of the $X_1$-type derivate only was identified on the silica gel chromatogram. Free phenol and 5 : 8-quinone were obtained on the alumina column.

Experiments were then made with liver homogenate fortified by the addition of crude UDPGA prepared from guinea-pig liver (Dutton and Storey, 1954).

A 10 per cent mouse liver homogenate was prepared in 0·154 M-KCl containing $3{\cdot}2 \times 10^{-4}$ M $KHCO_3$. Incubations were then carried out at 37° C. with 30 c.c. homogenate to which was added 20 c.c. 0·2 M phosphate buffer pH 7·4, 25 mg. ascorbic acid in 12 c.c. water, 6 c.c. 0·3 M $MgCl_2$, 5 c.c. of colloidal 8-benzpyrenol containing 1·25 mg., 35 c.c. of crude UDPGA extract and 50 c.c. water. In a control experiment the UDPGA extract was substituted by 35 c.c. water. After 2 hours the incubates were extracted as described for the *in vivo* tissue.

The extract of the fortified homogenate yielded a wide bright blue fluorescent zone at the surface of the silica gel which was absent from the control column.

Little blue fluorescent material was removed from this zone on prolonged development with amyl alcohol. On elution with ethanol it was identified as the $X_2$-type metabolite by its conversion to 8-benzpyrenol both by $\beta$-glucuronidase and prolonged acid hydrolysis.

Considerable amounts of the 5 : 8-quinone were obtained from both the experimental and control incubates, possibly due to oxidation of the phenol by ascorbic acid (cf. Warren, 1943).

It is concluded therefore that formation of the $X_2$-type metabolite is dependent upon the presence of UDPGA and in this respect conforms to accepted knowledge concerning the synthesis of glucuronides.

*Sulphate synthesis in liver homogenate*

Following on from preliminary work on the synthesis of sulphates in liver homogenate (De Meio and Tkacz, 1950, 1952 ; De Meio and Wizerkaniuk, 1952 ; De Meio, 1952) it was shown by Bernstein and McGilvery (1952) and De Meio, Wizerkaniuk and Fabiani (1953) that the enzyme system is located in the high speed supernatant fraction of the homogenate and requires ATP and $Mg^{++}$ ions for activity.

In the present experiments a 10 per cent mouse liver homogenate in Tyrode buffer (60 c.c.) was fortified with 20 c.c. 0·154 M $MgSO_4$ and 10 mg. ATP (sodium salt) and incubated with 0·5 mg. of 8-benzpyrenol at 37° C. for 2 hours. In a control expriment ATP was omitted from the mixture.

After extraction the $X_1$-type derivative only was identified with any certainty in the control experiment. The ATP fortified homogenate however, yielded a much increased yield of the $X_1$-type derivative and a smaller zone of what appeared to be the $X_2$-type. On elution with ethanol however, this latter fraction exhibited predominantly the absorption spectrum of the $X_1$-type metabolite suggesting that, in the absence of much $X_2$-type derivative, the $X_1$-type is only partially removed from silica gel by amyl alcohol. This provides a possible explanation for the appearance of the chromatogram noted for tissue extracts of animals injected with 8-benzpyrenol (this paper) and 3 : 4-benzpyrene (Harper, 1958*b*).

After elution with ethanol, further blue fluorescent material was removed from the $X_2$-type zone by 1 : 1 ethanol-phosphate buffer but insufficient was obtained for characterisation.

Contrary to the findings of De Meio, Wizerkaniuk and Fabiani (1953) with respect to the synthesis of phenyl-sulphate in liver homogenate, replacement of the ATP by a mixture of adenylic and $\alpha$-ketoglutaric acids did not increase the yield of the $X_1$-type metabolite.

Experiments using fortified " microsome free " supernatant fractions of the homogenate have given varied results. An initial preparation gave excellent yields of the $X_1$-type metabolite only. In repetitive experiments however, the account of the $X_1$-type derivative varied considerably being practically zero on occasions. No explanation for this behaviour can be offered but it is possibly due to technical errors inherent in the establishment of the *in vitro* sulphate synthesising system.

No definite conclusion can therefore be arrived at from these experiments but it is considered significant that, on the occasions when evidence of increased metabolism has been obtained, the $X_1$-type derivative has been present as the major metabolite.

*Metabolism in cat liver slices*

Certain evidence exists to suggest that cat liver does not synthesise glucuronides (Hartiala, 1955 ; Dutton and Greig, 1957). In addition no glucuronides could be isolated from the urine of cats fed various glucuronidogenic substances (Professor R. T. Williams, private communication quoted by Dutton and Greig, 1957.)

Fifty grammes young cat liver were sliced and incubated in 100 c.c. Tyrode buffer containing 0·3 M $MgSO_4$ and 1·25 mg. 8-benzpyrenol with oxygenation.

After extraction a considerable amount of the $X_1$-type metabolite was obtained on the chromatogram. Once again however, a blue fluorescence was associated with the $X_2$-type zone. Its ethanol eluate was only weakly fluorescent and its absorption spectrum was more like that of the $X_1$-type metabolite. Also, unlike the $X_2$-type, it did not give a coloured ether extract in the naphthoresorcinol test for glucuronic acid.

If the evidence that cats do not produce glucuronides is correct then this result is in accordance with the view that the $X_2$-type metabolite is the glucuronide. The possibility of the $X_1$-type being so is eliminated.

*The biosynthesis of $^{35}S$-labelled $X_1$-type metabolite*

The involvement of inorganic sulphate in the synthesis of ethereal sulphates receives considerable support from the studies of Laidlaw and Young (1953) who observed the excretion of radioactive 2-naphthylsulphuric and 2-amino-1-naphthylsulphuric acids after the simultaneous injection of $^{35}S$-labelled inorganic sulphate and 2-naphthol and 2-naphthylamine respectively.

A similar synthesis of $^{35}S$-labelled phenylsulphate in liver slices was reported by Sato, Suzuki and Yoshikawa (1953).

In the present experiments 30 g. of mouse liver were sliced and incubated at 37° C. for 3 hours in 150 c.c. Tyrode buffer containing 1·25 mg. 8-benzpyrenol and 0·9 mc. $Na_2\,{}^{35}SO_4$ with oxygenation. In a control experiment 8-benzpyrenol was omitted from the mixture.

The slices were then extracted and the distribution of radioactivity determined in the $X_1$- and $X_2$-type derivatives and corresponding fractions from the control experiment.

The association of radioactivity with the metabolites was then determined after heating their aqueous solutions under nitrogen in 0·1 N-HCl for 15 minutes, saturating with ammonium sulphate and extracting with ether. Under these conditions 8-benzpyrenol was removed from the $X_1$-type solution whereas the $X_2$-type derivative was extracted practically unchanged. The ether extracts were then transferred to 2 c.c. water and their activity determined.

The results of these experiments are shown in Table II.

TABLE II

| | Counts per minute | |
|---|---|---|
| | Ethanol eluates transferred to 4 c.c. water | Ether extracts of acid hydrolysates transferred to 2 c.c. water |
| 8-Benzpyrenol $X_1$ . . | 1190 | 67 |
| Control $X_1$-zone . . | 60 | 38 |
| 8-Benzpyrenol $X_2$ . . | 469 | 76 |
| Control $X_2$-zone . . | 328 | 68 |

The results indicate a close association of radioactivity with the $X_1$-type metabolite.

The fact that the activities of the $X_2$-type zones from the experimental and control runs are of the same order and the fact that this activity is removed under conditions which leave the metabolite unchanged suggests that the activity is due to the presence of $^{35}S$-labelled material unconnected with the metabolism of the phenol.

*Summary of results*

The following facts are considered to establish the identity of the $X_2$-type metabolite as 8-benzpyrenylglucuronic acid :

(1) Its chromatographic behaviour (cf. Barlow, 1957).
(2) Its relative stability to acid hydrolysis (cf. Bray and Thorpe, 1954).
(3) The colour reaction with naphthoresorcinol reagent.
(4) Its hydrolysis to 8-benzpyrenol by $\beta$-glucuronidase and the inhibition of this hydrolysis by $10^{-2}$ M boiled saccharate solution.
(5) Its synthesis in liver homogenate fortified with crude UDPGA.
(6) The apparent inability of cat liver slices to effect its synthesis.

The following facts are considered to establish the identity of the $X_1$-type metabolite as 8-benzpyrenylsulphuric acid:

(1) Its chromatographic behaviour (cf. Barlow, 1957).
(2) Its instability to acid (cf. Bray and Thorpe, 1954).
(3) Its hydrolysis to 8-benzpyrenol by takadiastase.
(4) Its synthesis in liver homogenate fortified with ATP and the less consistent results of the " microsome free " supernatant experiments.
(5) The synthesis of $^{35}S$-labelled $X_1$-type metabolite by liver slices in the presence of $Na_2\,{}^{35}SO_4$.

*Metabolism of the $F_1$-phenol*

The small quantity of the phenol available has severely limited its experimental use so that it has not been possible to confirm the results by repetition.

The general appearance of the opened abdominal cavity was similar to that after 8-benzpyrenol. The intensity of the fluorescence was much reduced by comparison however, presumably due to the much lower dosage level.

*Gall bladder, duodenum and small intestine*

The combined gall bladder, duodenum and small intestine were extracted as described for 8-benzpyrenol. On chromatographic separation similar blue fluorescent $X_1$- and $X_2$-type fractions were obtained.

The ethanol eluate of the $X_1$-type zone possessed only weak absorption but pronounced maxima were present at 298 and 378–380 m$\mu$ with inflections at 362, 369 and 406–410 m$\mu$ (Table I). On acidification with hydrochloric acid and gentle warming under nitrogen it was converted back into the $F_1$-phenol as shown by the appearance of the 418 m$\mu$ band in the absorption spectrum and the fluorescent colour change from blue to green in sodium hydroxide.

Strongly absorbing background material prevented possible characterisation of the $X_2$-type metabolite by absorption spectroscopy but, on transfer to water and incubation with $\beta$-glucuronidase, an ether extract exhibited the characteristic absorption spectrum of the $F_1$-phenol.

In view of this evidence and by analogy with the behaviour of 8-benzpyrenol it is concluded that the $F_1$-phenol is similarly conjugated with sulphuric and glucuronic acids.

### RE-EXAMINATION OF THE METABOLISM OF 3 : 4-BENZPYRENE AND PYRENE

### (a) 3 : 4-*Benzpyrene*

Identification of the $X_1$-type metabolite of 8-benzpyrenol as the sulphate conjugate is in direct conflict with previous evidence suggesting the presence of a $\beta$-glucuronosidic linkage in the $X_1$ metabolite derived from 3 : 4-benzpyrene. The discovery that 8-benzpyrenylsulphuric acid is instantly decomposed by acid and ether suggested a possible explanation for this discrepancy for in the previous series of experiments, after incubation with $\beta$-glucuronidase, the solutions were routinely acidified with hydrochloric acid to facilitate removal of the phenol by ether. It was essential therefore to repeat those experiments under the same conditions used for the 8-benzpyrenyl metabolites.

*Effect of acid.*—On acidification of the aqueous solution of the metabolites free $F_1$-phenol was immediately extractable from the $BPX_1$ solution with ether. The $BPX_2$ metabolite tended to form a scum at the interface but spectroscopically appeared to be unchanged.

The $BPX_2$ was then heated in 10 N-HCl at 100° C. under nitrogen for 2 hours. An ether extract of the hydrolysate was yellow in colour with a bright blue fluorescence. It absorption spectrum was predominantly that of the $F_1$-phenol but attenuation of the 395 and 418 m$\mu$ bands suggested the presence of some 8-benzpyrenol. This was verified on transfer to hexane when the 396 and 419–420 m$\mu$ bands of 8-benzpyrenol appeared in the $F_1$ spectrum.

In a further experiment the ether extract of the $BPX_2$ hydrolysate was transferred to xylene and chromatographed on a column of silica gel over alumina when the phenolic fraction was obtained as a bright yellow/green fluorescent zone on the alumina. The absorption spectrum of the ethanol eluate from this zone was predominantly that of the $F_1$-phenol but an additional band at 425–426 m$\mu$ indicated the presence of 8-benzpyrenol.

Thus, behaviour so far was similar to that of the 8-benzpyrenyl conjugates. A further phenomenon was observed however in the case of the $BPX_2$ on acid hydrolysis. The xylene filtrate from the silica gel/alumina chromatogram invariaby possessed a blue/violet fluorescence. On evaporating the filtrate to dryness and dissolving the residue in ethanol the characteristic absorption spectrum of 3 : 4-benzpyrene was obtained.

*Tests for glucuronic acid.*—Solutions of the metabolites were heated with naphthoresorcinol reagent as described for 8-benzpyrenol. Similar colorations were obtained on extraction with ether suggesting the presence of a glucuronide moiety in $BPX_2$ only.

*Effect of $\beta$-glucuronidase.*—After incubation with $\beta$-glucuronidase a trace amount only of the $F_1$-phenol was removed from the $BPX_1$ solution by direct extraction with ether. A similar trace was obtained from the boiled enzyme control.

The $BPX_2$ incubate however, yielded a bright blue fluorescent ether extract which was not obtained from the boiled enzyme control. The presence of $F_1$-phenol and a smaller amount of 8-benzpyrenol in the extract was determined by absorption spectroscopy after transfer to hexane.

*Effect of takadiastase.*—Little blue fluorescent material was extracted from the $BPX_2$ solution by ether after incubation with takadiastase. The $BPX_1$ solution however, yielded a bright blue fluorescent extract exhibiting the characteristic absorption spectrum of the $F_1$-phenol. This contrasted markedly with the weak blue fluorescent extract when the boiled enzyme was used.

*Nature of $BPX_1$ and $BPX_2$.*—The above results invalidate previous conclusions as to the nature of the $X_1$ metabolite of 3 : 4-benzpyrene but confirm the presence of a $\beta$-glucuronosidic linkage in the $X_2$ metabolite.

In view of this evidence, and by analogy with the behaviour of the 8-benzpyrenyl metabolites, it is concluded that $BPX_2$ is a complex mixture containing at least three derivatives, the glucuronide of $F_1$, the glucuronide of 8-benzpyrenol and an acid-decomposable benzpyrene precursor.

The behaviour of $BPX_1$ is in accordance with it being the sulphate conjugate of $F_1$ only as 8-benzpyrenol has not, so far, been identified by absorption spectroscopy as a product of its hydrolysis. The possibility of a small amount of 8-benzpyrenol being present in the $F_1$ cannot entirely be ruled out however, for in a parallel case, Conney, Miller and Miller (1957) have reported that a methylated metabolite possessing the characteristic absorption spectrum of 8-methoxybenzpyrene did in fact contain some of the 10-methoxyderivative.

Attempted fractionation of $BPX_1$ by partition chromatography has failed to establish the presence of 8-benzpyrenyl-sulphuric acid. Such a result is not surprising perhaps in view of the very similar properties of, and the close spectral relationship between, the metabolites and the fact that only a small amount of free 8-benzpyrenol appears in the caecum and large intestine during the first few hours following intravenous injection of 3 : 4-benzpyrene.

It seemed possible that more direct evidence for its presence would be afforded by an investigation of the $BPX_1$ obtained after the intraperitoneal injection of the hydrocarbon for, under these conditions, the amount of 8-benzpyrenol initially appearing in the faeces is increased (Weigert and Mottram, 1946). Accordingly the $X_1$ metabolite was isolated from the small intestines of mice killed 6 hours after the intraperitoneal injection of 1 mg. of 3 : 4-benzpyrene. There was no marked difference in its absorption spectrum but, on mild acid hydrolysis, a mixture of $F_1$ and 8-benzpyrenol was obtained as shown by the presence of doublets in the absorption spectrum at 392 and 396 m$\mu$ and 415 and 419–420 m$\mu$ in hexane as solvent (Harper, 1958*b*).

This fact therefore is considered to provide good evidence for the metabolic formation of 8-benzpyrenylsulphuric acid from 3 : 4-benzpyrene.

Attempts to fractionate the $BPX_2$ complex were equally unsuccessful in that separation into the respective glucuronides was not achieved. It was established however, that the hydrocarbon precursor is concentrated in a narrow tan coloured surface zone of the chromatogram and its presence appears to be associated with the appearance of a 346 m$\mu$ maximum in the $BPX_2$ absorption spectrum (Fig. 3). Successive lower fractions of the zone possessed similar absorption spectra but slight variations in the position of the absorption maxima were noted. The significance of these shifts has not been established but it is suggestive of the presence of more than one metabolite.

The absorption spectrum of $BPX_2$ obtained in the filtrate after development of the chromatogram with ethanol is also recorded here (Fig. 3) as it is considered to establish the fully aromatic benzpyrenoid configuration of the metabolite.

FIG. 3.—Absorption spectra in ethyl alcohol. $BPX_2$ fractions: 1. Ethanol filtrate. 2. Top surface zone. (For explanation see text.)

(b) *Pyrene*

In a previous study of the metabolism of pyrene two intermediate metabolites were isolated (Harper, 1958*a*). One was identified as 3-pyrenylglucuronic acid and appeared to be the major metabolite excreted in the bile. The other, signified as PX, was only obtained from the *in vivo* liver but was formed, together with the glucuronide and free phenol, from pyrene in isolated liver slices. It was rapidly decomposed to 3-hydroxypyrene by acid and in view of the similar behaviour found for 8-benzpyrenylsulphuric acid the possibility of it being 3-pyrenylsulphuric acid has been tested.

Separation of the pyrene metabolites in the small intestine into $X_1$ and $X_2$-type fractions was carried out as described for 8-benzpyrenol.

The $X_2$ zone yielded a blue/violet fluorescent solution exhibiting the characteristic absorption spectrum of 3-pyrenylglucuronide. This was confirmed by its hydrolysis to 3-hydroxypyrene both by β-glucuronidase and acid. The presence of a small amount of free pyrene in the acid hydrolysate was also established by xylene extraction followed by chromatography on silica gel over alumina. This is in accordance with the presence of an acid-labile precursor of pyrene previously reported in the faeces (Harper, 1957).

The $X_1$ zone yielded a pale blue fluorescent solution exhibiting the characteristic absorption spectrum of PX. The PX remained unaffected on incubation with β-glucuronidase but was rapidly hydrolysed to 3-hydroxypyrene by takadiastase.

It is concluded therefore that PX is 3-pyrenylsulphuric acid and that, in like manner to 8-benzpyrenol, 3-hydroxypyrene is eliminated in the bile in conjugation with both glucuronic and sulphuric acids.

### OTHER HYDROCARBONS

In the case of two other hydrocarbons, chrysene and 20-methylcholanthrene, the same pattern of biliary excretion has been observed. $X_1$- and $X_2$-type derivatives have been isolated from the duodenum and small intestine and these have undergone hydrolysis to phenolic compounds in the caecum and large intestine.

An acid-decomposable precursor of 20-methylcholanthrene has also been found in its $X_2$ complex.

### DISCUSSION

Although the absolute identification of the metabolites of 3 : 4-benzpyrene must eventually lie in their individual isolation and characterisation, with chemical synthesis as the final check, the results of these experiments are considered to provide good evidence for the presence of sulphate and glucuronide conjugates of the $F_1$-phenol and 8-benzpyrenol in the mixture of metabolites excreted via the gall bladder. The formation of such compounds was of course to be anticipated from the metabolic studies carried out by Conney, Miller and Miller (1957). These workers investigated the metabolism of 3 : 4-benzpyrene in fortified liver homogenate and identified 8-benzpyrenol, 10-benzpyrenol and the $F_1$-phenol as major products of oxidation. As 10-benzpyrenol has also been identified as an excretion product of 3 : 4-benzpyrene in the faeces (Berenblum and Schoental, 1946) a similar sequence of conjugation and hydrolysis is to be anticipated but the present experiments have yielded no evidence on this point. In this connection it is perhaps significant that Chalmers (1956), using more refined paper partition chromatographic methods, has isolated a metabolic fraction possessing an absorption spectrum suggestive of 10-methoxybenzpyrene and giving a positive test for glucuronic acid.

The fact that the $F_1$-phenol has not been detected as an excretion product of 8-benzpyrenol in these experiments suggests that it is formed as a separate entity during the primary oxidation of 3 : 4-benzpyrene in the liver. The findings of Conney, Miller and Miller (1957) previously referred to are consistent with this view. If this is the case then a possible metabolic sequence would be as in Fig. 4.

FIG. 4.—1. Oxidation by microsome/TPNH system of tissue. 2. Conjugation with glucuronic and sulphuric acids. 3. Hydrolysis in caecum and large intestine. 4. Partial oxidation to quinones.

The unknown factors in this scheme are the identity of the $F_1$-phenol and the mechanism by which the sulphate conjugates are hydrolysed in the gut. No new evidence has been forthcoming on the former point but the acid-labile nature of the sulphate conjugates suggests that possible pH changes down the gut could play a part in their hydrolysis, as suggested by Weigert and Mottram (1946). The other possibility is hydrolysis by arylsulphatase activity, and on this point no conclusive information appears to exist. Dodgson, Spencer and Thomas (1953) reported that the intestine possessed but little arylsulphatase activity but this result was based on the activity of only small pieces of the intestine, the exact location of which was not stated. Indeed, in view of the fact that a spectrophotometric method of assay was used, it is unlikely that the highly coloured caecal contents would be amenable to such an investigation.

Obviously further work must be done to establish this point but it is perhaps significant that certain strains of bacteria have been found to possess arylsulphatase activity (e.g. Whitehead, Wildy and Engbaek, 1953).

The failure of Gutmann and Wood (1950) to obtain evidence of $^{35}S$-labelled metabolites of 3 : 4-benzpyrene from the urine of animals previously dosed with $^{35}S$-labelled L-cystine and DL-methionine appears to be at variance with the biosysnthesis of radioactive 8-benzpyrenylsulphuric acid reported here. It must be borne in mind however that only a relatively small percentage (17 per cent according to Heidelberger and Weiss, 1951) of injected 3 : 4-benzpyrene is eliminated in the urine. The present experiments suggest that only a small fraction of this can be attributed to the presence of the sulphate conjugates and of course further hydrolysis to the phenols presumably takes place during the collection period. It is doubtful therefore whether such a small increase in activity would be detected in an experiment where an appreciable standard deviation existed. In the same experiment the failure to obtain evidence of increased excretion of radioactivity in the faeces can be explained by resorption of the sulphate radicals after hydrolysis of the conjugates in the caecum. That the colon is an active site of sulphate fixation is shown by the work of Pasternak, Kent and Davies (1958), and Pasternak and Kent (1958).

The identification of the $X_1$ and $X_2$ metabolites of 3 : 4-benzpyrene as sulphate and glucuronide conjugates respectively can now be correlated with the findings of Calcutt and Payne (1954) with respect to the intracellular distribution of the metabolites in the liver.

The sulphate synthesising system is located in the supernatant fraction of the homogenate and it was in this fraction only that the $X_1$ metabolite was detected.

The system responsible for glucuronide synthesis on the other hand, is located in the microsomal fraction (Dutton and Storey, 1954 ; Strominger, Kalckar, Axelrod and Maxwell, 1954), but a lower level of activity is also associated with the mitochondrial and nuclear fractions (Dutton, 1956). Calcutt and Payne obtained the $X_2$ metabolite from all fractions but Calcutt (private communication) considers the bulk of the metabolite to be located in the microsomes.

The identification of 3-pyrenyl glucuronic and sulphuric acids as biliary excretion products of pyrene and their hydrolysis to 3-hydroxypyrene in the caecum and large intestine suggests that this metabolic sequence may be a common pattern of excretion amongst the polycyclic hydrocarbons. This is borne out by the isolation of $X_1$- and $X_2$-type derivatives of chrysene and 20-methylcholanthrene. Chalmers (1957) has also reported the excretion of conjugated metabolites of anthracene in the bile. The possibility of a similar sequence for 1 : 2 : 5 : 6-dibenzanthracene is suggested by the unchanged excretion in the faeces of the unidentified phenolic metabolite from the rabbit after its reinjection into mice (Dobriner, Rhoads and Lavin, 1942).

The formation of acid-labile precursors of the hydrocarbons may also be a common factor. The excretion of such compounds has already been reported for a variety of hydrocarbons (Chang and Young, 1943), and a similar behaviour has now been observed for pyrene, 3 : 4-benzpyrene and 20-methylcholanthrene.

SUMMARY

1. The excretions of 8-benzpyrenol and the $F_1$ metabolite of 3 : 4-benzpyrene have been investigated after intravenous injection in the mouse. The same pattern of excretion has been observed as that arising from injection of the parent hydrocarbon i.e., the elimination of X-type derivatives in the bile and their hydrolysis back to the phenols in the caecum and large intestine.

2. The $X_1$- and $X_2$-type metabolites of 8-benzpyrenol have been identified as the sulphate and glucuronide conjugates of the phenol respectively and similar structures are proposed for the metabolites derived from the $F_1$-phenol.

3. A re-examination of the metabolism of 3 : 4-benzpyrene has invalidated previous conclusions as to the nature of the $X_1$ metabolite ($BPX_1$) but has confirmed the presence of glucuronic acid in the $X_2$ metabolite ($BPX_2$).

4. Evidence has been obtained that $BPX_2$ is in fact a complex mixture containing the glucuronides of $F_1$ and 8-benzpyrenol together with an acid-labile precursor of 3 : 4-benzpyrene.

5. The behaviour of $BPX_1$ is in accordance with it being the sulphate conjugate of $F_1$ only but evidence for the presence of 8-benzpyrenylsulphuric acid in the $BPX_1$ has been obtained after the intraperitoneal injection of the hydrocarbon.

6. A possible metabolic sequence to account for these facts is proposed.

7. A re-examination of the metabolism of pyrene has been made. The hitherto unidentified metabolite isolated from the liver has now been identified as 3-pyrenylsulphuric acid and found to be an excretion product in the bile together with 3-pyrenylglucuronic acid and an acid-labile precursor of the hydrocarbon.

8. Chrysene and 20-methylcholanthrene have yielded similar $X_1$- and $X_2$-type metabolites undergoing hydrolysis to phenolic products in the caecum. It is suggested that this may be a common pattern of excretion for the polycyclic hydrocarbons.

I am much indebted to Mr. F. S. Stewart for his advice on the handling of radioactive compounds and to Parke Davis, Co. Ltd. for their gift of takadiastase.

### REFERENCES

Barlow, J. J.—(1957) *Biochem. J.*, **65**, 34 P.

Berenblum, I., Crowfoot, D., Holiday, E. R. and Schoental, R.—(1943) *Cancer Res.*, **3**, 151.

*Idem* and Schoental, R.—(1946) *Ibid.*, **6**, 699.

Bernstein, S. and McGilvery, R. W.—(1952) *J. biol. Chem.*, **198**, 195.

Boyland, E. and Wiltshire, G. H.—(1953) *Biochem. J.*, **53**, 636.

BRAY, H. G. AND THORPE, W. V.—(1954) " Analysis of Phenolic Compounds of Interest in Metabolism " in ' Methods of Biochemical Analysis ', Vol. 1, ed. D. Glick. London (Interscience Publishers Ltd.).
CALCUTT, G. AND PAYNE, S.—(1954) *Brit. J. Cancer*, **8**, 554, 710.
CHALMERS, J. G.—(1956) *Ibid.*, **10**, 787.—(1957) *Rep. Brit. Emp. Cancer Campgn.*, **35**, 302.
CHANG, L. H. AND YOUNG, L.—(1943) *Proc. Soc. exp. Biol. N.Y.*, **53**, 126.
CONCHIE, J. AND LEVVY, G. A.—(1957) *Biochem. J.*, **65**, 389.
CONNEY, A. H., MILLER, E. C. AND MILLER, J. A.—(1957) *J. biol. Chem.*, **228**, 753.
COOK, J. W., LUDWICZAK, R. S. AND SCHOENTAL, R.—(1950) *J. chem. Soc.*, 1112.
DE MEIO, R. H.—(1952) *Acta physiol. lat.-amer.*, **2**, 195.
*Idem* AND TKACZ, L.—(1950) *Arch. Biochem.*, **27**, 242.—(1952) *J. biol. Chem.*, **195**, 175.
*Idem* AND WIZERKANIUK, M.—(1952) Résumés des communications, 2e Congrès International de Biochimie, Paris, 306.
*Idem*, WIZERKANIUK, M. AND FABIANI, E.—(1953) *J. biol. Chem.*, **203**, 257.
DOBRINER, K., RHOADS, C. P. AND LAVIN, G. I.—(1942) *Cancer Res.*, **2**, 95.
DODGSON, K. S., SPENCER, B. AND THOMAS, J.—(1953) *Biochem. J.*, **53**, 452.
DUTTON, G. J.—(1956) *Ibid.*, **64**, 693.
*Idem* AND GREIG, C. G.—(1957) *Ibid.*, **66**, 52 P.
*Idem* AND STOREY, I. D. E.—(1954) *Ibid.*, **57**, 275.
GUTMANN, H. R. AND WOOD, J. L.—(1950) *Cancer Res.*, **10**, 8, 701.
HARPER, K. H.—(1957) *Brit. J. Cancer*, **11**, 499.—(1958*a*) *Ibid.*, 12, 116.—(1958*b*) *Ibid.*, **12**, 121.
HARTIALA, K. J. V.—(1955) *Ann. Med. exp. Fenn.*, **33**, 239.
HEIDELBERGER, C. AND WEISS, S. M.—(1951) *Cancer Res.*, **11**, 885.
KARUNAIRATNAM, M. C. AND LEVVY, G. A.—(1949) *Biochem. J.*, **44**, 599.
LAIDLAW, J. C. AND YOUNG, L.—(1953) *Ibid.*, **54**, 142.
LEVVY, G. A.—(1948) *Ibid.*, **42**, 2.—(1952) *Ibid.*, **52**, 464, 690.
PASTERNAK, C. A. AND KENT, P. W.—(1958) *Ibid.*, **68**, 452.
*Idem*, KENT, P. W. AND DAVIES, R. E.—(1958) *Ibid.*, **68**, 212.
ROBINSON, D., SMITH, J. N., SPENCER, B. AND WILLIAMS, R. T.—(1952) *Ibid.*, **51**, 202.
SATO, T., SUZUKI, T. AND YOSHIKAWA, H.—(1953) *J. Biochem. (Tokyo)*, **40**, 663.
STOREY, I. D. E.—(1950) *Biochem. J.*, **47**, 212.
*Idem* AND DUTTON, G. J.—(1955) *Ibid.*, **59**, 279.
STROMINGER, J. L., KALCKAR, H. M., AXELROD, J. AND MAXWELL, E. S.—(1954) *J. Amer. chem. Soc.* **76**, 6411.
WARREN, F. L.—(1943) *Biochem. J.*, **37**, 338.
WEIGERT, F. AND MOTTRAM, J. C.—(1946) *Cancer Res.*, **6**, 97.
WHITEHEAD, J. E. M., WILDY, P. AND ENGBAEK, H. C.—(1953) *J. Path. Bact.*, **65**, 451.


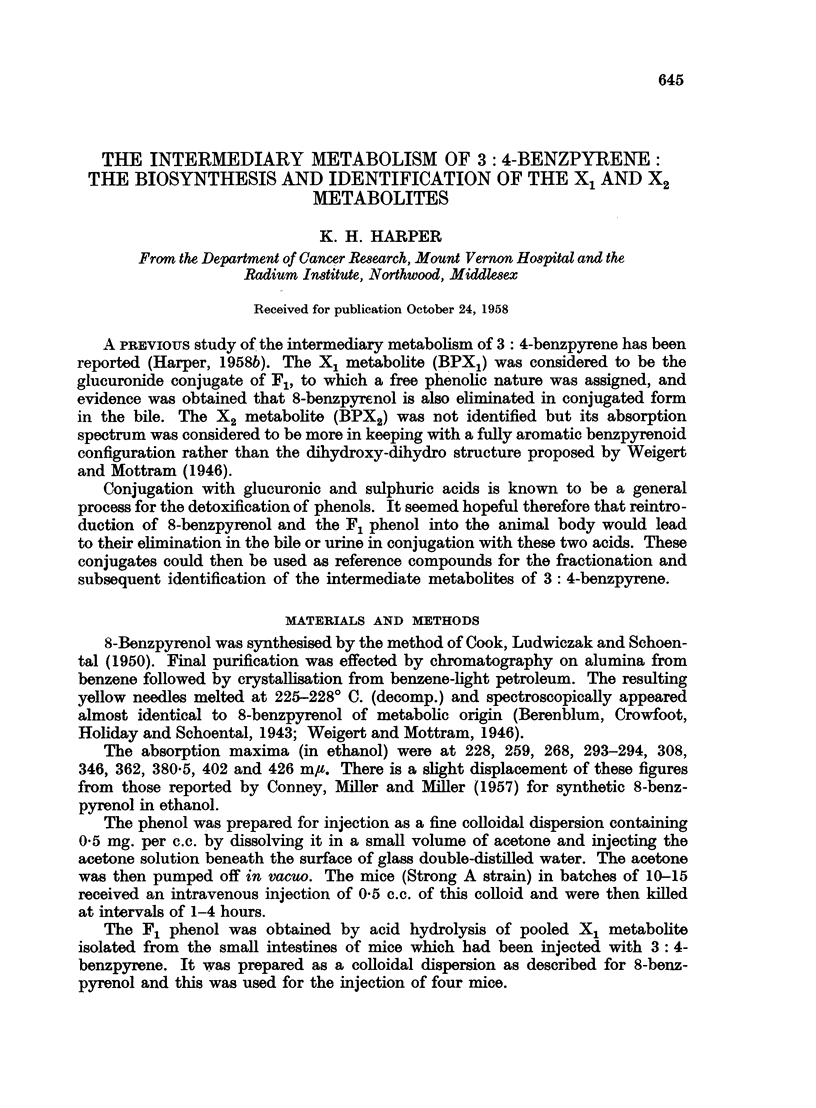

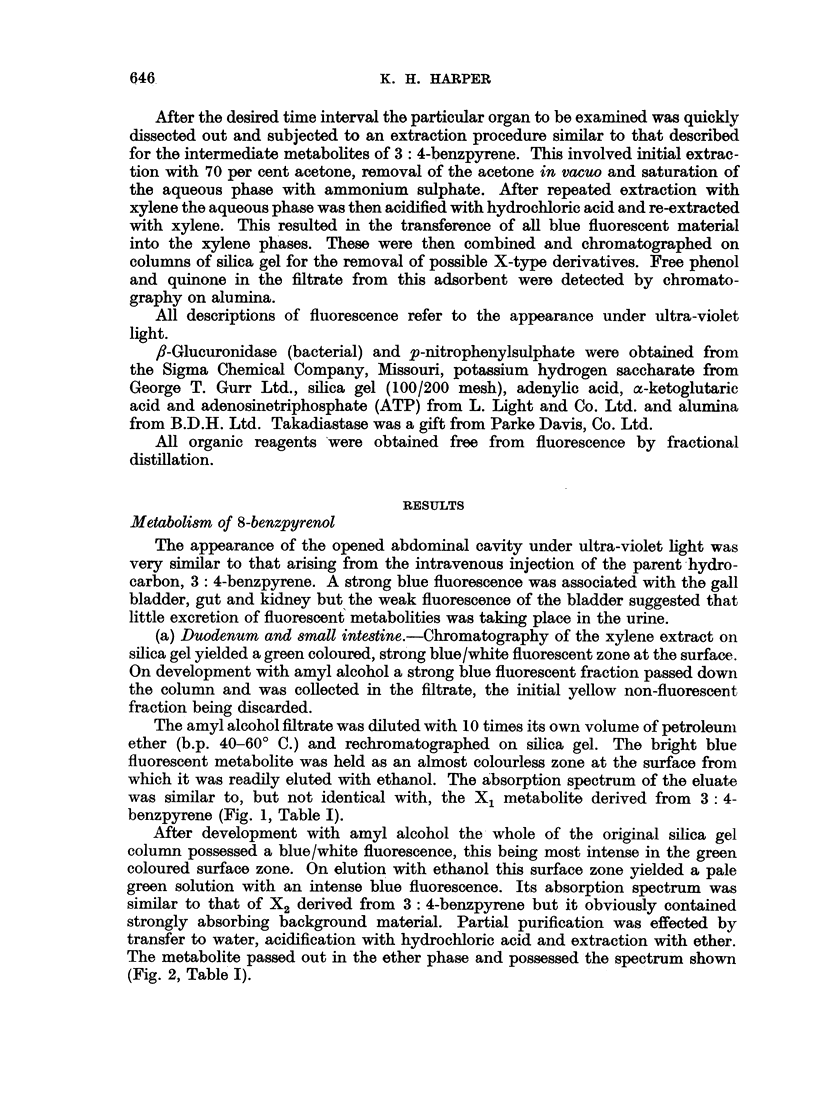

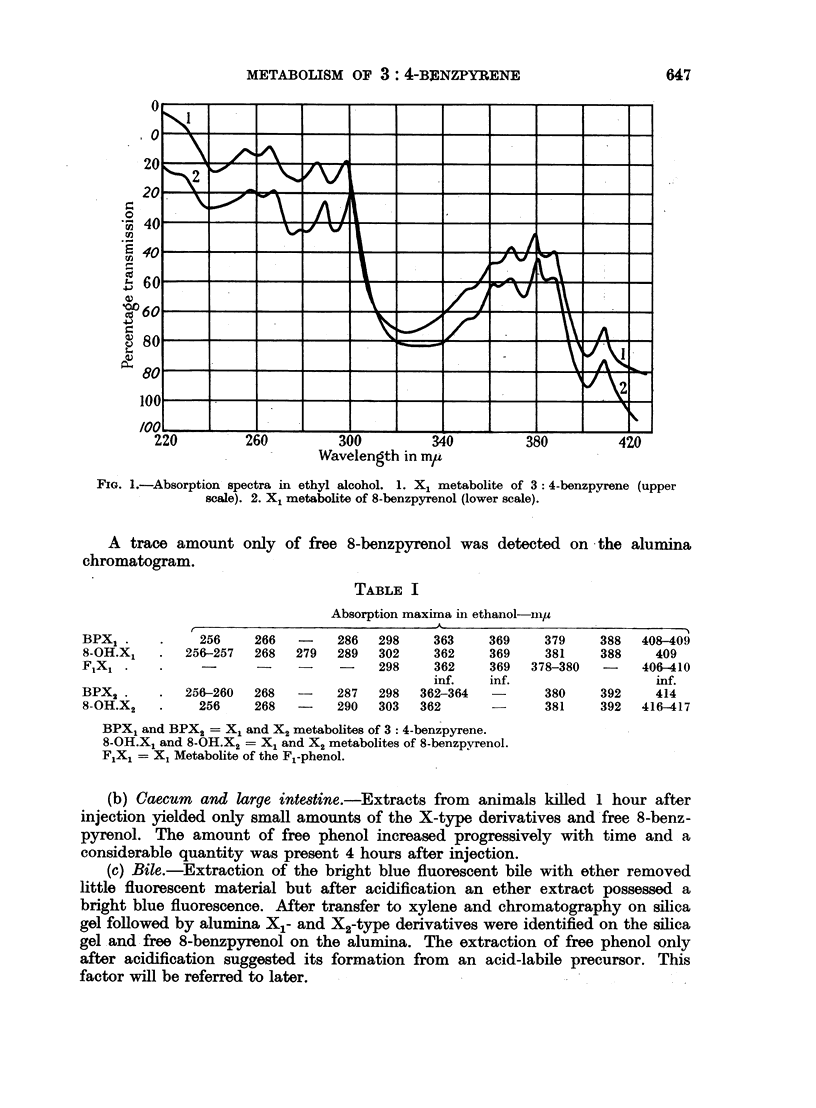

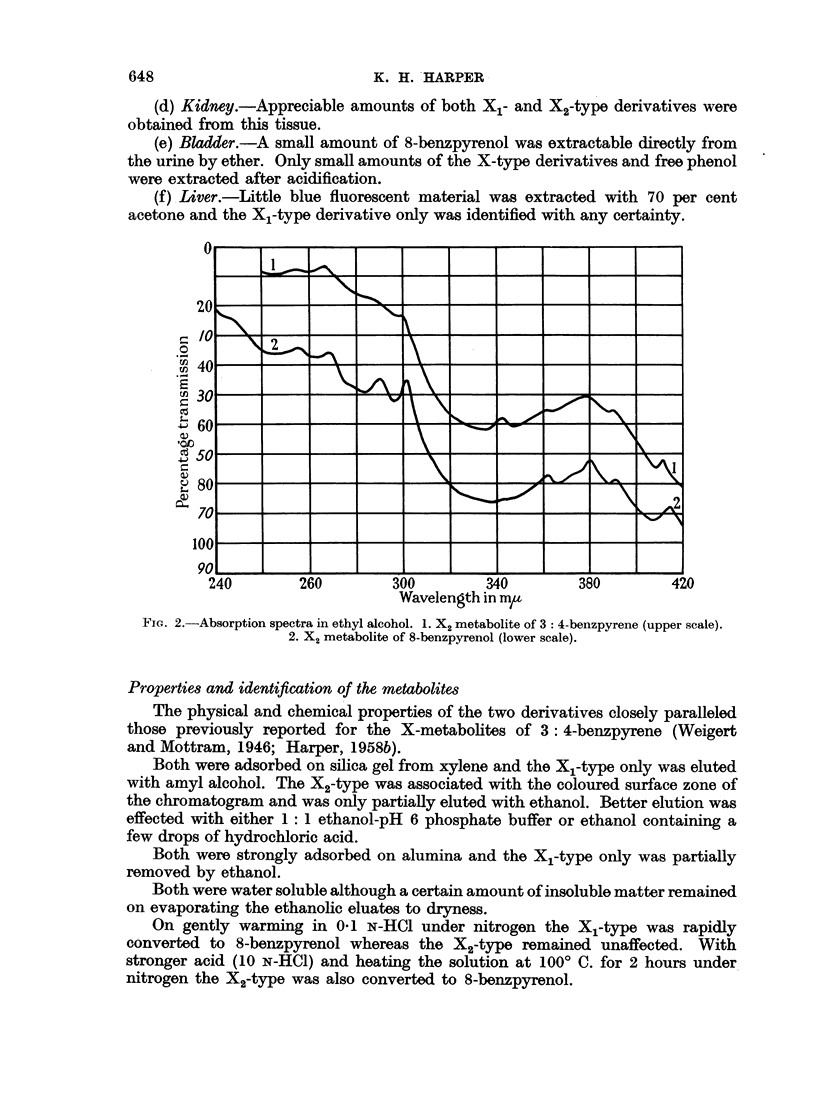

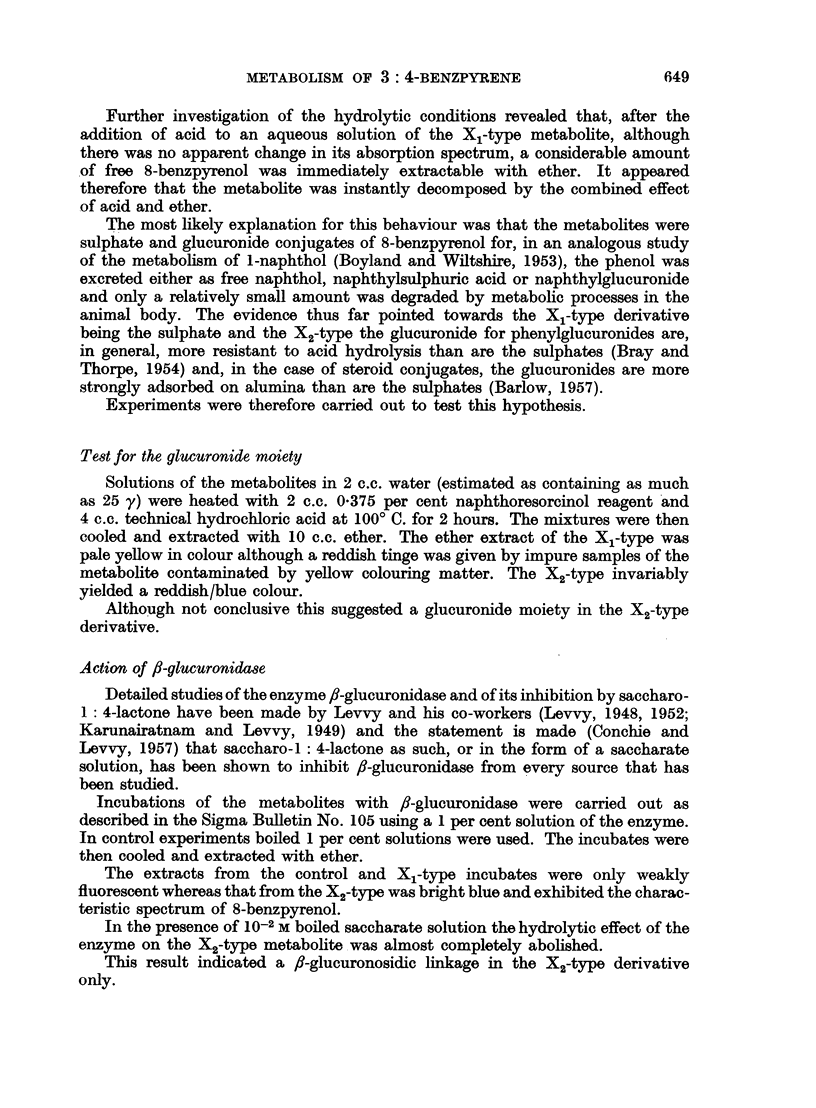

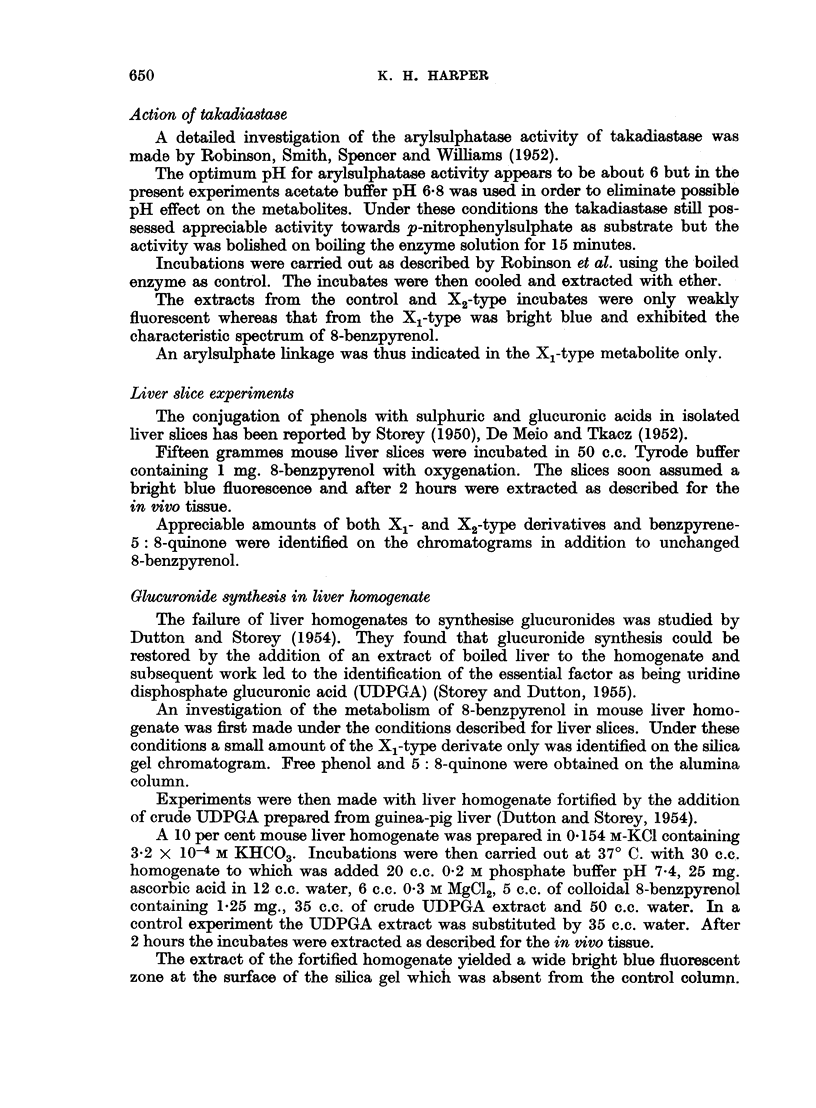

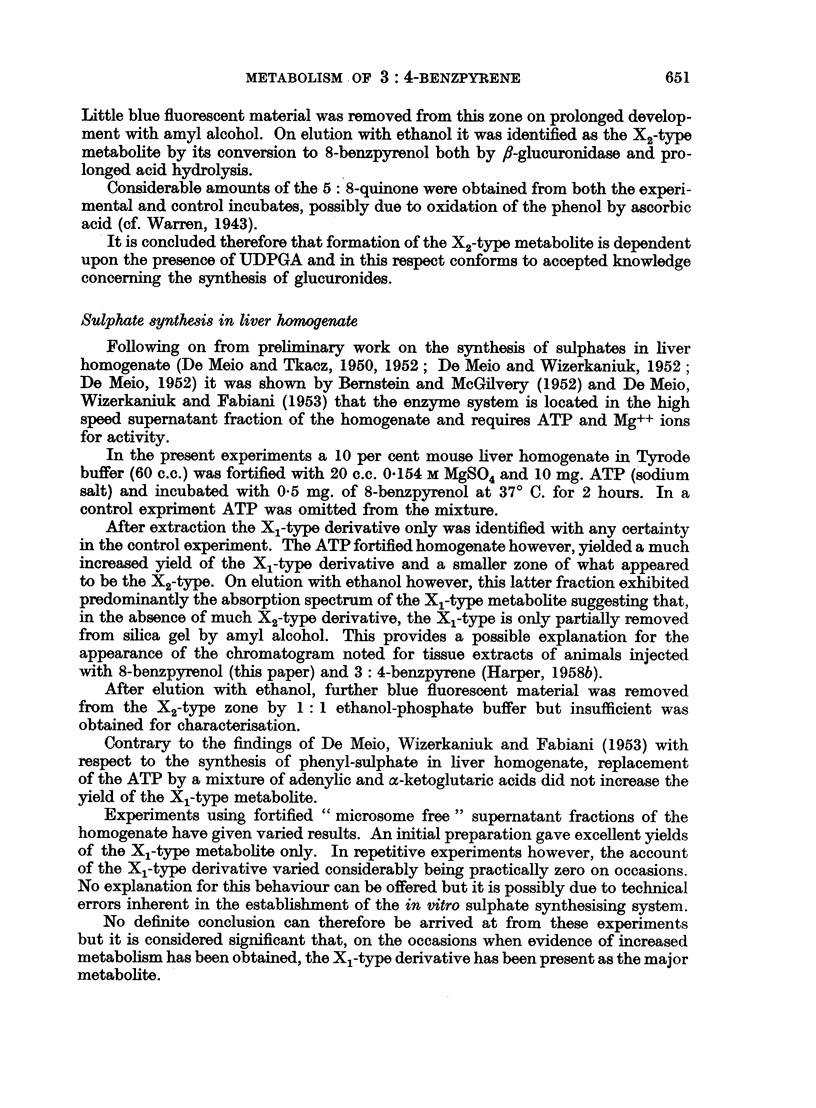

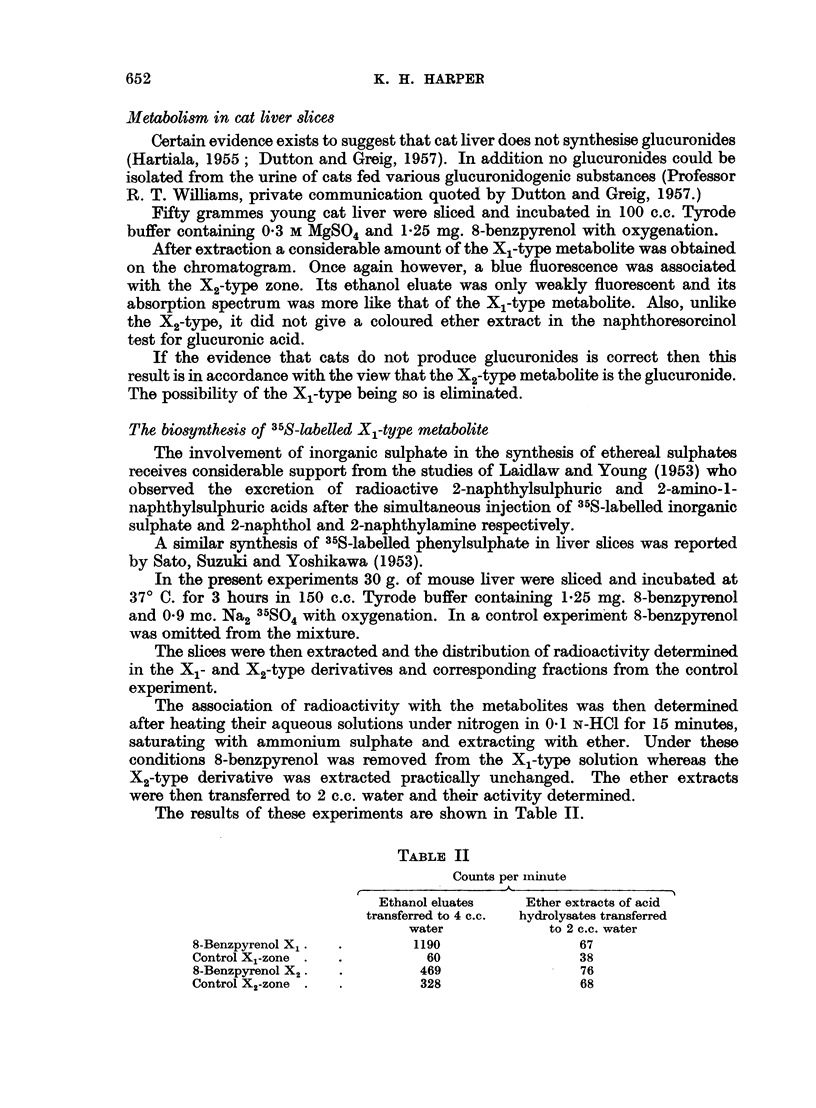

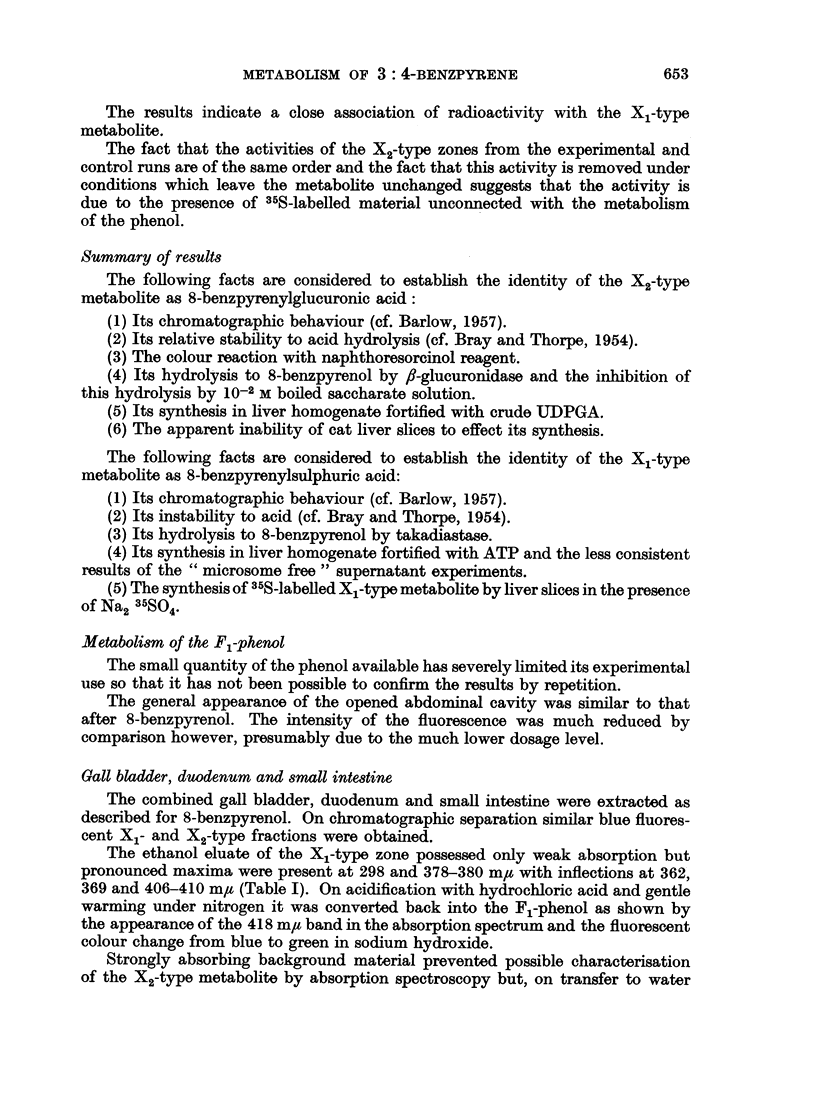

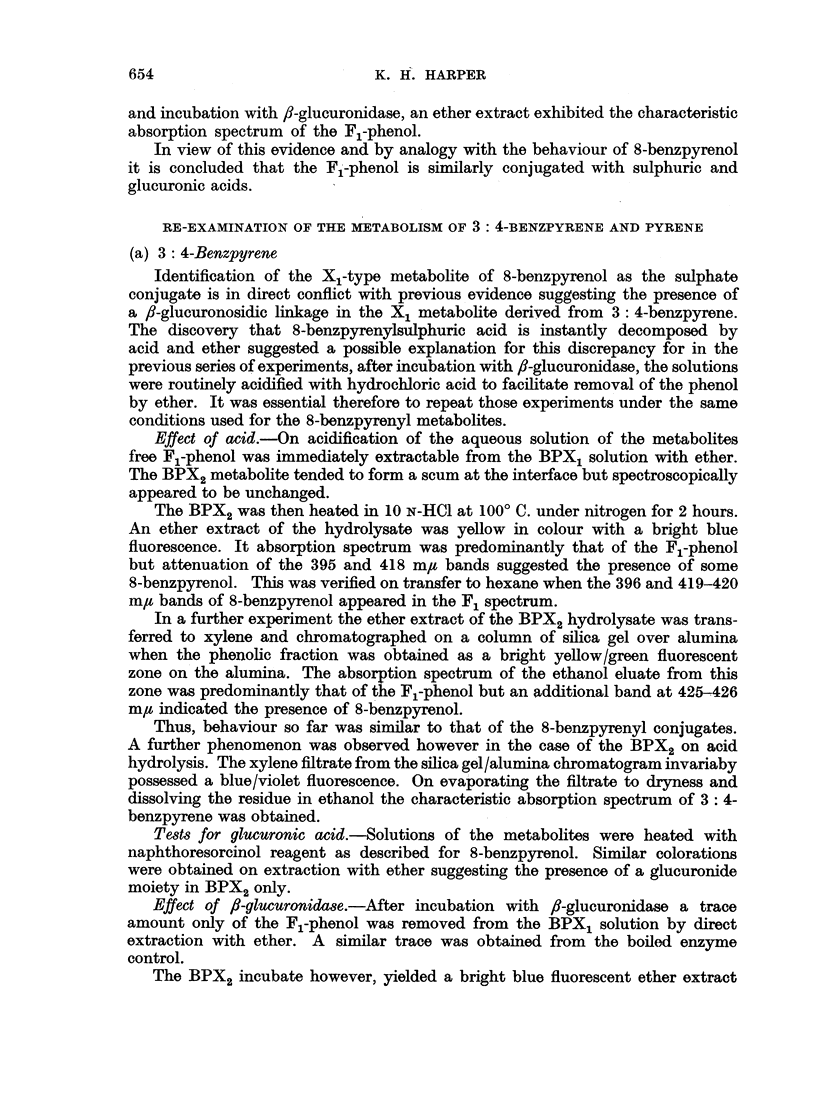

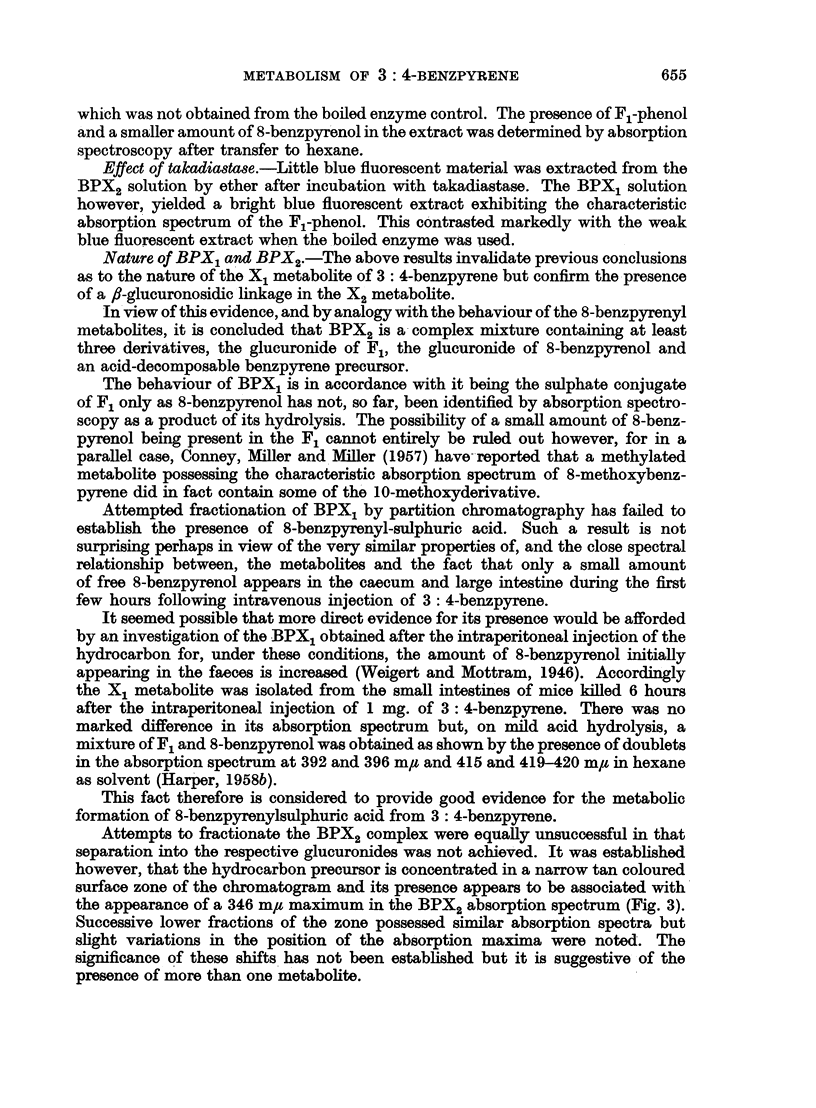

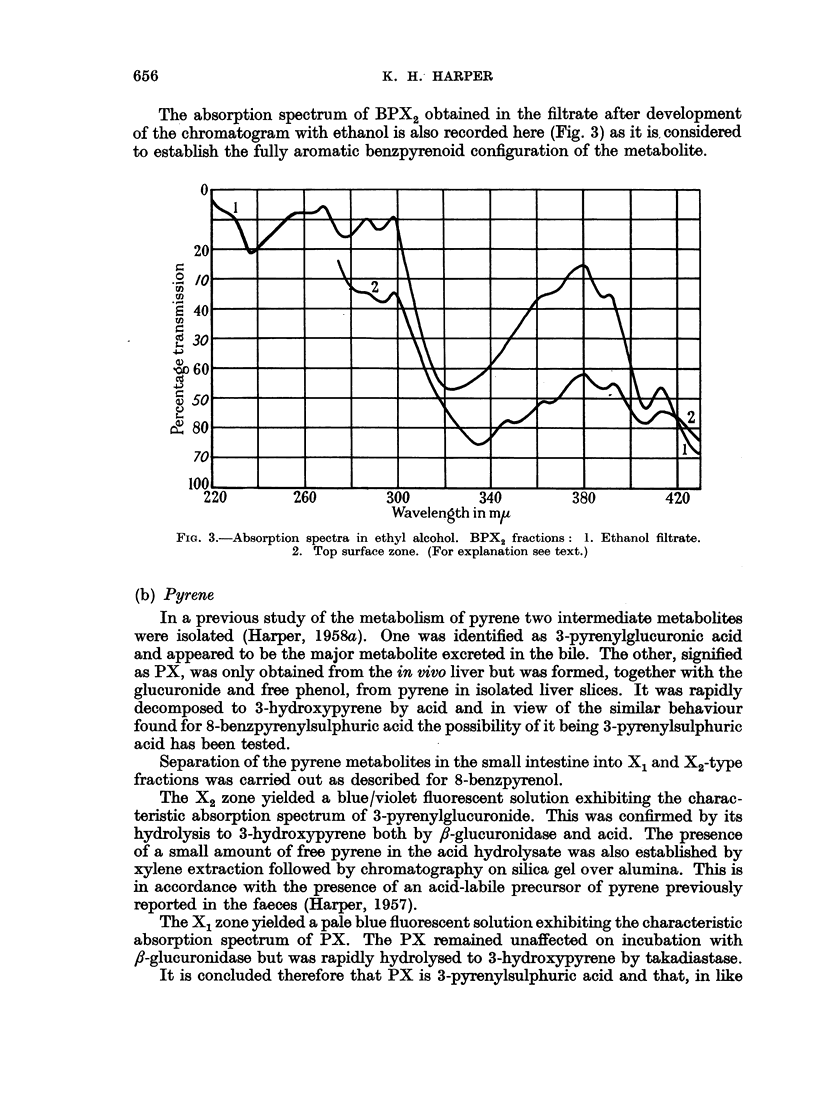

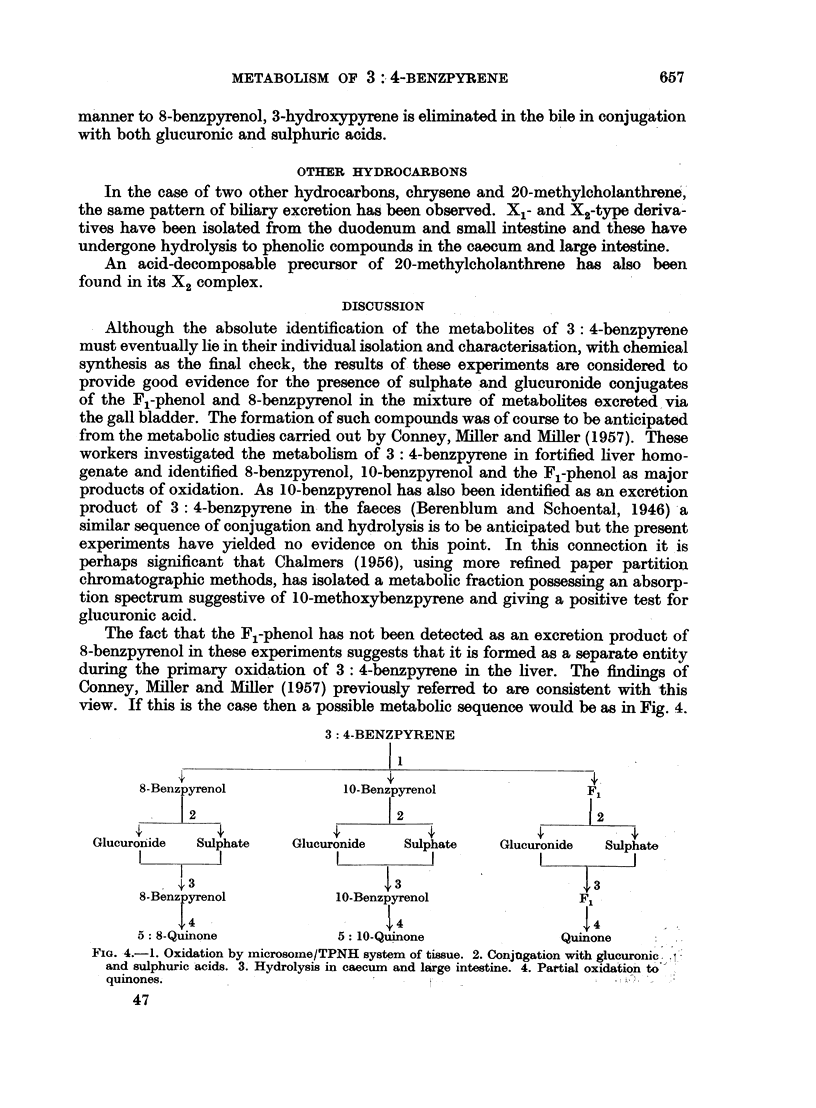

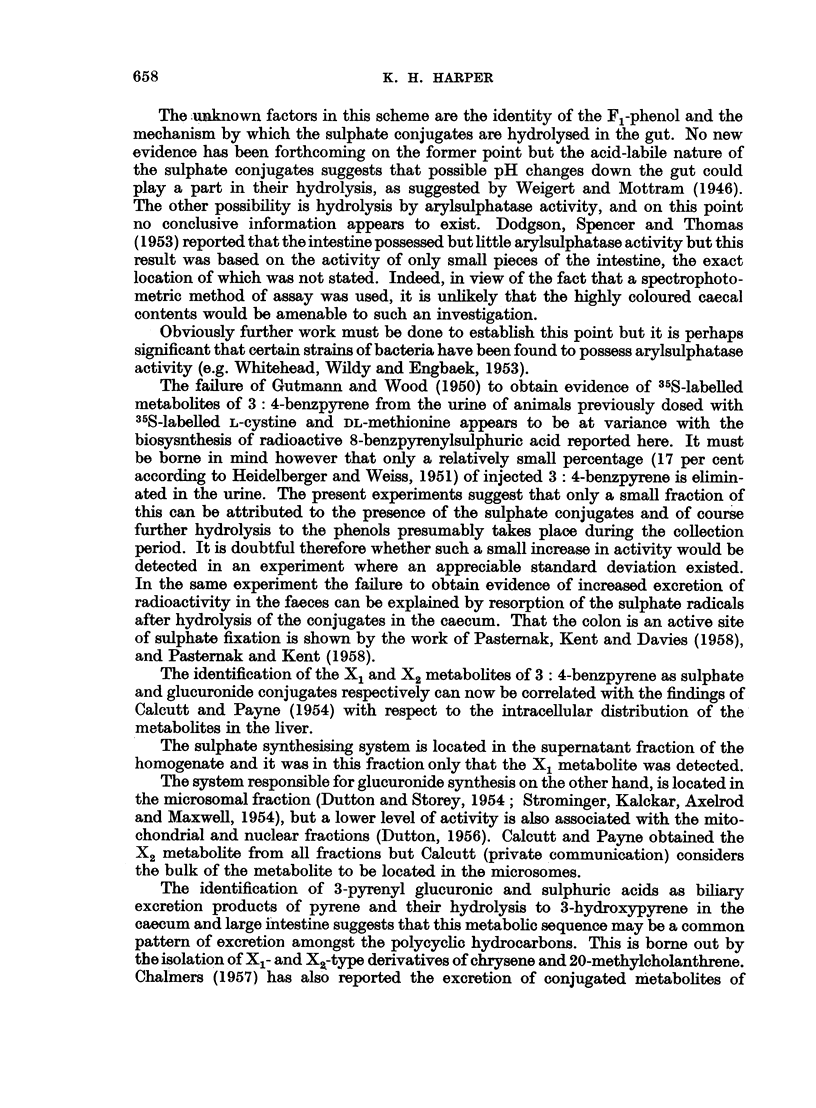

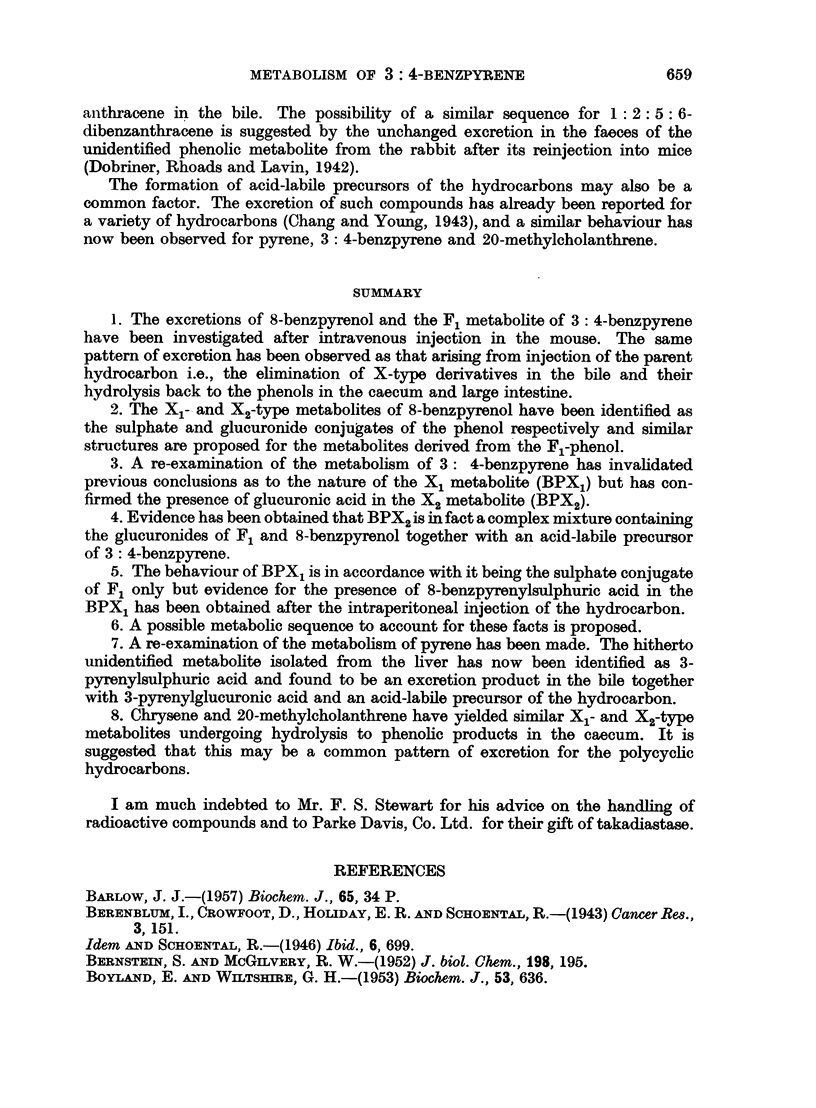

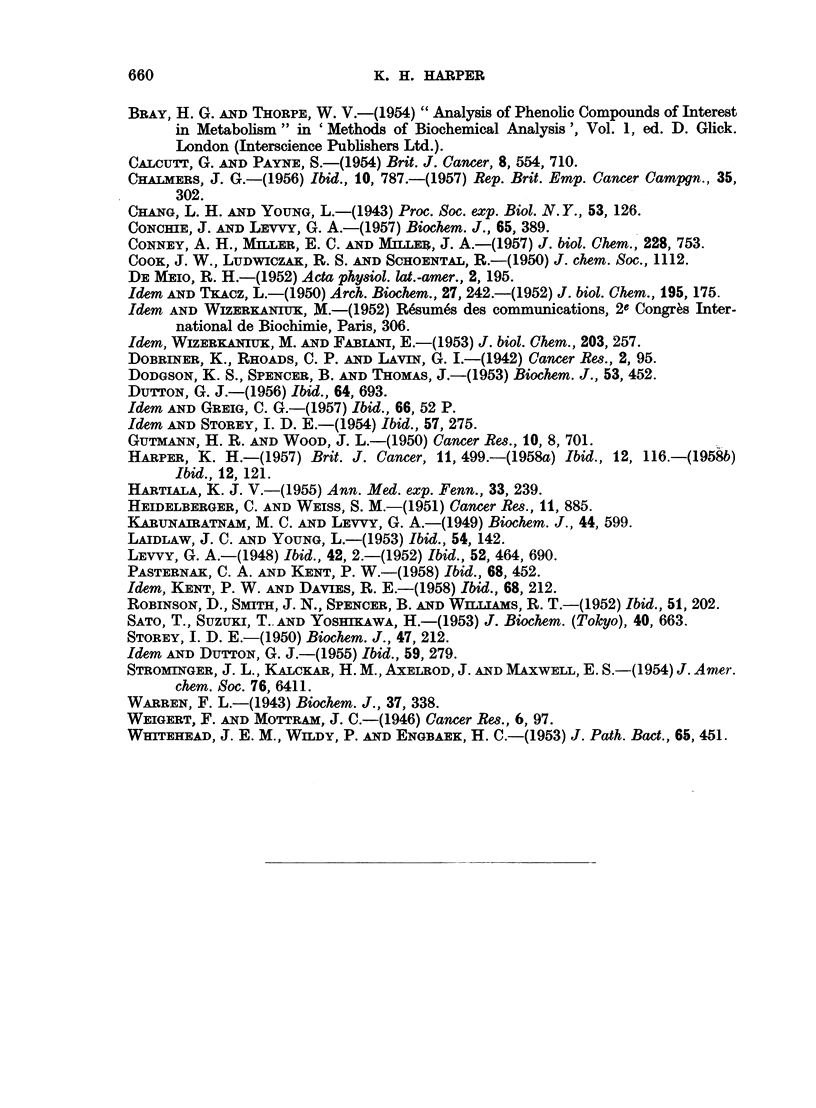


## References

[OCR_00940] BERNSTEIN S., MCGILVERY R. W. (1952). The enzymatic conjugation of m-aminophenol.. J Biol Chem.

[OCR_00943] BOYLAND E., WILTSHIRE G. H. (1953). Metabolism of polycylic compounds. 7. The metabolism of naphthalene, 1-naphthol and 1:2-dihydroxy-1:2-diphydronaphthalene by animals.. Biochem J.

[OCR_00952] CALCUTT G., PAYNE S. (1954). The intracellular metabolism of 3:4 benzpyrene; benzpyrene metabolites from rats and their sites of formation in rat liver.. Br J Cancer.

[OCR_00959] CONNEY A. H., MILLER E. C., MILLER J. A. (1957). Substrate-induced synthesis and other properties of benzpyrene hydroxylase in rat liver.. J Biol Chem.

[OCR_00961] DE MEIO R. H. (1952). Sintesis del p-nitrofenilsulfato por el hígado de rata.. Acta Physiol Lat Am.

[OCR_00963] DE MEIO R. H., TKACZ L. (1952). Conjugation of phenol by rat liver slices and homogenates.. J Biol Chem.

[OCR_00969] DE MEIO R. H., WIZERKANIUK M., FABIANI E. (1953). Role of adenosinetriphosphate in the enzymatic synthesis of phenyl sulfate.. J Biol Chem.

[OCR_00973] DUTTON G. J. (1956). Uridine diphosphate glucuronic acid as glucuronyl donor in the synthesis of ester, aliphatic and steroid glucuronides.. Biochem J.

[OCR_00979] GUTMANN H. R., WOOD J. L. (1950). The urinary excretion of mercapturic acids after administration of bromobenzene and 3,4-benzpyrene.. Cancer Res.

[OCR_00985] HARTIALA K. J. (1955). Studies on detoxication mechanisms. III. Glucuronide synthesis of various organs with special reference to the detoxifying capacity of the mucous membrane of the alimentary canal.. Ann Med Exp Biol Fenn.

[OCR_00987] HEIDELBERGER C., WEISS S. M. (1951). The distribution of radioactivity in mice following administration of 3,4-benzpyrene-5-C14 and 1,2,5,6-dibenzanthracene-9,10-C14.. Cancer Res.

[OCR_00989] Karunairatnam M. C., Levvy G. A. (1949). The inhibition of beta-glucuronidase by saccharic acid and the role of the enzyme in glucuronide synthesis.. Biochem J.

[OCR_00992] LAIDLAW J. C., YOUNG L. (1953). Biochemical studies of toxic agents. IV. A study of ethereal sulphate formation in vivo using radioactive sulphur.. Biochem J.

[OCR_00993] PASTERNAK C. A., KENT P. W. (1958). Biosynthesis of intestinal mucins. 2. Incorporation of [35S] sulphate by guinea-pig colon in vitro.. Biochem J.

[OCR_00995] PASTERNAK C. A., KENT P. W., DAVIES R. E. (1958). Biosynthesis of intestinal mucins. 1. Survey of incorporation of [35S] sulphate by isolated gastrointestinal tissues.. Biochem J.

[OCR_00997] ROBINSON D., SMITH J. N., SPENCER B., WILLIAMS R. T. (1952). Studies in detoxication. XXXXIII. A study of the arylsulphatase activity of takadiastase towards some phenolic ethereal sulphates.. Biochem J.

[OCR_00999] STOREY I. D. E. (1950). The synthesis of glucuronides by liver slices.. Biochem J.

[OCR_01011] WHITEHEAD J. E., WILDY P., ENGBAEK H. C. (1953). Arylsulphatase activity of mycobacteria.. J Pathol Bacteriol.

[OCR_01007] Warren F. L. (1943). Aerobic oxidation of aromatic hydrocarbons in the presence of ascorbic acid: The reaction with anthracene and 3:4-benzpyrene.. Biochem J.

